# Construction of a Prognostic Model for KIRC and Identification of Drugs Sensitive to Therapies - A Comprehensive Biological Analysis Based on m6A-Related LncRNAs

**DOI:** 10.3389/fonc.2022.895315

**Published:** 2022-06-02

**Authors:** Dian Xia, Qi Liu, Songbai Yan, Liangkuan Bi

**Affiliations:** ^1^ Department of Urology, The Second Hospital of Anhui Medical University, Hefei, China; ^2^ Department of Oncology, The First Affiliated Hospital of Anhui Medical University, Hefei, China

**Keywords:** kidney renal clear cell carcinoma, m6A, LncRNA, prognosis, immune response, bioinformatics

## Abstract

As one of the common malignancies in the urinary system, kidney cancer has been receiving explorations with respect to its pathogenesis, treatment and prognosis due to its high morbidity, high mortality and low drug efficiency. Such epigenetic modifications for RNA molecules as N6-methyladenosine (m6A) usher in another perspective for the research on tumor mechanisms, and an increasing number of biological processes and prognostic markers have been revealed. In this study, the transcriptome data, clinical data and mutation spectrum data of KIRC in the TCGA database were adopted to construct an m6A-related lncRNA prognostic model. Besides, the predictive ability of this model for clinical prognosis was evaluated, and some compounds sensitive to therapies for KIRC were screened. The findings of this study demonstrate that this effective and stable model has certain clinical application value.

## Introduction

Renal cell carcinoma (RCC) is the most common malignancy in the kidneys. There are about 210,000 new patients with this disease worldwide each year, accounting for 2%-3% of all cancer cases. Kidney renal clear cell carcinoma (KIRC) or clear cell renal cell carcinoma (ccRCC) is a main histological subtype of RCC, accounting for 80%-90% of the total number of RCC patients. There is a poor prognosis for patients with KIRC, which seriously affects their life and health (1). Although surgical treatment is effective in the treatment of patients with kidney cancer at an early stage, the recurrence and metastasis may occur in as many as 30% of patients after radical surgery, who have unfavorable survival and prognosis (2). Generally, the patient with metastatic renal cell carcinoma (mRCC) cannot be cured, with the median survival being only 18 months and a low 5-year survival rate. In recent years, some patients with kidney cancer have benefited from the immune checkpoint inhibitors, especially the programmed death receptor-1 (PD-1) and its ligand (PD-L1) inhibitors (3). However, the overall effective rate of immunotherapies is less than 40%, and a considerable number of patients cannot benefit from immunotherapies (4). As per some analysis results, in addition to the low sensitivity of patients with kidney cancers to immunosuppressants, drug resistance in tumors is also a common reason for the decreased treatment efficiency. Therefore, the survival and prognosis of patients with kidney cancer can be effectively improved by exploring the important biological processes in the occurrence and development of kidney cancer and identifying drugs sensitive to tumor treatment.

N6-methyladenosine (m6A) refers to a methylation modification located on the 6th nitrogen atom of adenine. It often contains a conservative motif RRACH (R stands for A or G, H stands for A, C or U). It is the most common apparent modification of eukaryotic RNA and exists on various RNAs, such as messenger RNA (mRNA), long non-coding RNA (lncRNA), micro RNA (miRNA) and circular RNA (circRNA) (5). m6A modification is a dynamic and reversible equilibrium process, which is maintained by methyltransferase, demethylase and reader proteins (6). m6A methylation modification is involved in regulating the shear processing, nuclear translocation, degradation and translation of mRNAs, thus playing a decisive role in the entire life cycle of mRNA. In recent years, increasing attention has been paid to the relationship between m6A methylation modification and tumor occurrence and development. There is a significant difference in the total m6A modification levels between RCC cell lines and normal renal tubular epithelial cell lines; The major role of m6A methyltransferase has been confirmed by analyzing the correlation between 19 m6A regulatory factors (7). WTAP correlates with the expression of METTL3 and METTL14, which together affect the level of m6A modification (8). Besides, WTAP is the only transferase with a known interaction with other 5 m6A methyltransferases. It is significantly up-regulated in ccRCC, and its high expression is also related to the overall survival (OS) of ccRCC patients (9). Further, the high expression of m6A-related gene ALKBH5 positively correlates with tumor volume, TNM stage and poor prognosis of patients with kidney cancer (10). These findings indicate that m6A plays a vital role in KIRC.

The long non-coding RNA (lncRNA) is an RNA with a length of more than 200bp that cannot encode proteins, and it is extensively distributed in the nucleus and cytoplasm (11). In the previous, lncRNAs were thought to be the “noise” in the process of gene expression (12). However, DERRIEN et al. (13) found that lncRNAs are produced through a transcriptional pathway similar to that of the coding gene and have similar histone modifications, splicing patterns and exons/introns. LncRNAs are transcribed from either strand of the coding gene, and they can or not be polyadenylated (14). Currently, it has been confirmed in related studies that lncRNAs have a decisive role in RCC. WANG et al. (15) found that the lncRNA RP11-436H11.5 can be overexpressed in kidney cancer cells OSRC-2, the expression level of the oncogene BCL-W protein is elevated and cell invasion is also enhanced. After these cells are treated with the BCL-W inhibitor ATB-737, cell invasion is reduced; the inhibition is more pronounced at a higher concentration of ATB-737. Meanwhile, HE et al. (16) analyzed the tissue and plasma samples from 46 patients with RCC, and they found that lncRNA GIHCG increases significantly in the tissue and plasma samples of these patients (P<0.01). The lncRNA GIHCG in stage II-IV is significantly higher than that in stage I (P=0.028). Besides, the lncRNA GIHCG in Fuhrman G3-G4 is significantly higher than that in Fuhrman G1-G2 (P=0.032).

As an important modification molecule, m6A can not only affect the trimming, transport and degradation of miRNAs, lncRNAs and circRNAs, but also regulate the biological functions of various cells by modulating the expression products of lncRNAs to affect the pathological processes of various diseases, which has been demonstrated in many studies (17).

In this study, the expression profiles of 2876 lncRNAs and 23 m6A genes were extracted from The Cancer Genome Atlas (TCGA) dataset. Next, the m6A-related lncRNAs were identified by Pearson’s correlation analysis. Subsequently, an m6A-related lncRNA prognostic model was developed to predict the overall survival (OS) of patients with KIRC. Then, the publicly available drug sensitivity database was utilized to identify candidate drugs targeting this m6A-related lncRNA signature. After that, the correlation with responses to immunotherapies was explored. Finally, a nomogram was plotted to predict the OS of these patients.

## Materials and Methods

### Raw Data

The transcriptome RNA-seq data, corresponding clinical data and mutation data of KIRC cases were downloaded from the TCGA database (https://portal.gdc.cancer.gov/) with API v3.0.0. (Data Release 31.0 - October 29, 2021). The validation cohort is the expression profile data of renal cell carcinoma in ICGC database (https://dcc.icgc.org/), a total of 91 samples of renal cell carcinoma. After batch elimination of ICGC expression matrix and TCGA expression matrix based on combat method using R sva package, the expression level of model LncRNA in ICGC expression matrix was extracted.The optimal cutoff value determined by X-tile software was used as the threshold to divide patients into a high-risk group and a low-risk group in ICGC database.

### Selection of m6A Genes and m6A-Related lncRNAs

The profiles of lncRNAs and m6A genes were obtained from the TCGA database. According to previous studies (18, 19), the expression matrixes of 23 m6A genes were retrieved from the TCGA database, including the expression data of writers (METTL3,METTL14, METTL16,WTAP,VIRMA,ZC3H13,RBM15,RBM15B), readers (YTHDC1,YTHDC2,YTHDF1,YTHDF2,YTHDF3,HNRNPC,FMR1,LRPPRC,HNRNPA2B1,IGFBP1,IGFBP2,IGFBP3,RBMX), and erasers (ALKBH5 and FTO). The m6A-related lncRNAs were screened by Pearson’s correlation analysis, and 464 m6A-related lncRNAs were identified based on the criteria of |Pearson R| >0.4 and p <0.001.

### Establishment and Validation of the Risk Signature

The entire TCGA set was randomized as a training set and a test set. The training set was utilized to construct an m6A-related lncRNA model, and the entire set and the test set were utilized to validate this established model. **Table S1** shows the baseline characteristics of these two sets. There was no significant difference in clinical features between both datasets (p > 0.05). Combined with the survival data of patients with KIRC in TCGA, the prognosis of m6A-related lncRNAs was screened from 253 m6A-related lncRNAs in the TCGA dataset (p < 0.05). Besides, univariate Cox regression was used in this study (20). After LASSO Cox regression was conducted with the assistance of the R package glmnet (using the penalty parameter estimated by 10-fold cross-validation), it was found that 19 m6A-related lncRNAs were distinctly related to the OS of KIRC patients from TCGA datasets. In addition, multivariate Cox regression (21) was applied to analyze 19 m6A-related lncRNAs, and a 10-m6A-related lncRNA risk model was ultimately established. The following formula (22) was used to calculate the risk score: Risk score = coef (lncRNA1) × expr (lncRNA1) + coef (lncRNA2) × expr (lncRNA2) + ……+ coef (lncRNAn) × expr (lncRNAn). In this formula, coef represents the coefficient, coef (lncRNAn) represents the coefficient of lncRNAs related to survival, and expr (lncRNAn) represents the expression of lncRNAs. According to the median risk score, subgroups including the low- and high-risk groups were established (23).

### Functional Analysis

GO analysis was performed to identify the differentially expressed genes (DEGs) with the assistance of the R package clusterProfiler. The analysis threshold was determined by the p value. p <0.05 indicated that the functional comment was significantly enriched.

### Exploration of the Model in the Immunotherapeutic Treatment

The mutation data were evaluated and calculated with the assistance of the R package maftools. The TMB was measured according to tumor-specific mutated genes. Further, the TIDE algorithm was adopted to predict the likelihood of the immunotherapeutic response (20).

### PCA and Kaplan-Meier Survival Analysis

PCA was conducted on effective dimensionality reduction, model identification, and grouping visualization of high-dimensional data of the entire gene expression profiles, 23 m6A genes, 464 m6A-related lncRNAs, and risk model according to the expression patterns of the 10 m6A-related lncRNAs (24). Additionally, Kaplan-Meier survival analysis was also conducted to appraise diversities in the OS between both groups. The R packages survMiner and survival were adopted in this process.

### Exploration of Potential Compounds Targeting the m6A-Related lncRNA Model in Clinical Treatment

In an attempt to identify potential compounds in clinical treatment of KIRC patients, the IC50 of compounds obtained from the GDSC website in the TCGA project of the KIRC dataset was calculated. The R package pRRophetic was used to predict the IC50 of compounds obtained from the GDSC website in patients with KIRC.

### Independence of the m6A-Related lncRNA Model

Multivariate and univariate Cox regression analyses were conducted to test whether the prognostic pattern was a variable independent of other clinical features (age, gender, stage and grade) in the patients with KIRC (25).

### Establishment and Verification of a Predictive Nomogram

The predictive ability of the nomogram and other predictors (age, gender, stage, grade and risk score) for the 1-, 3-, and 5-year OS was established. The correction curves based on the Hosmer-Lemeshow test were adopted to illustrate the uniformity between the practical outcome and model prediction outcome.

## Results

### Identification of m6A-Related LncRNAs in Patients With KIRC

**Figure 1:**
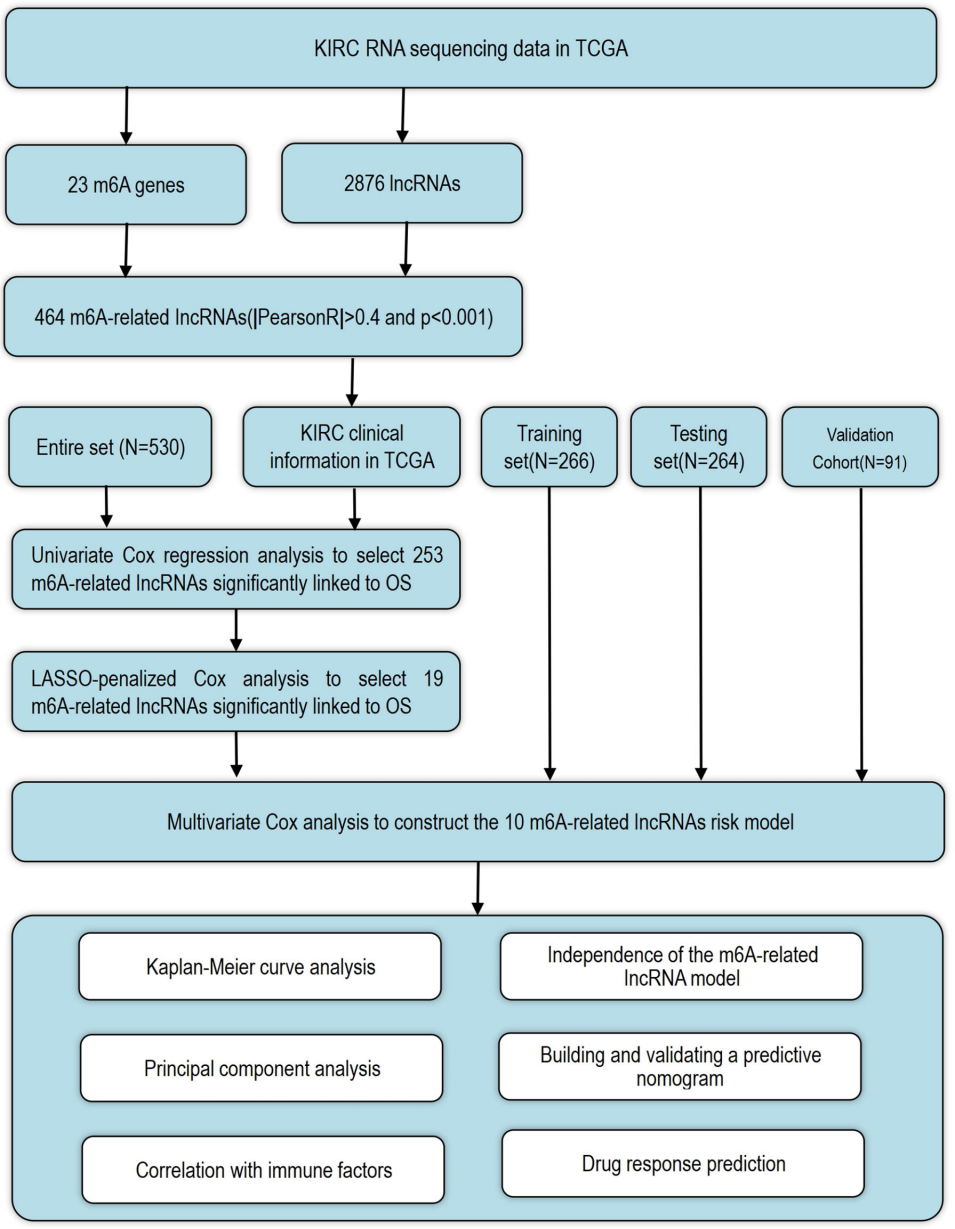
The workflow of the Research.

**Figure 2:**
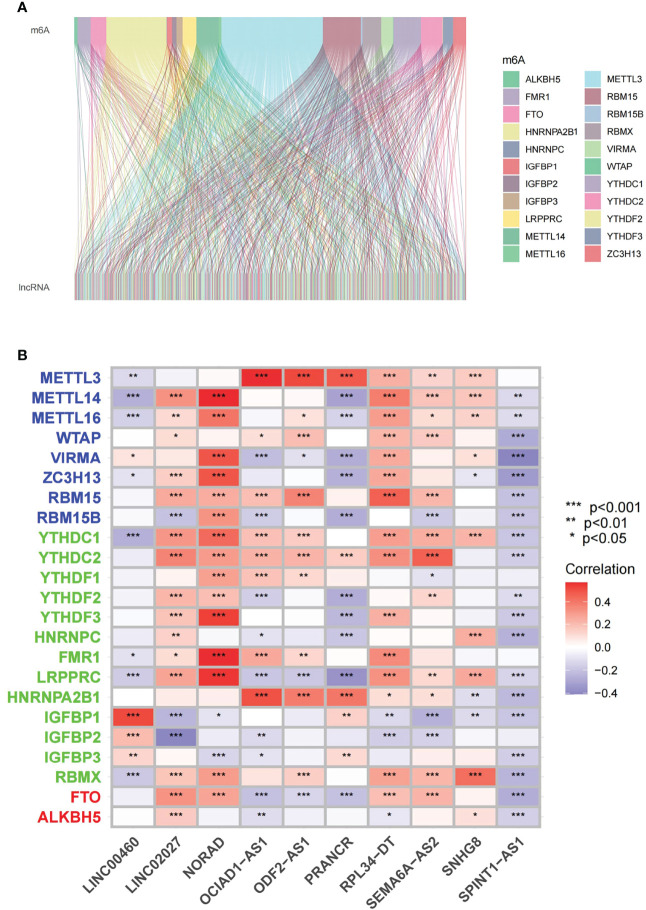
Identification of m6A-related lncRNAs in KIRC patients **(A)** Sankey relational diagram for 23 m6A genes and m6A-related lncRNAs. **(B)** Heatmap for the correlation between 23 m6A genes and the 10 prognostic m6A-related lncRNAs. *p < 0.05, **p < 0.01, ***p < 0.001.

The detailed workflow for the risk model construction and subsequent analyses is shown in **Figure 1**. The matrix expression of 23 m6A genes and 2876 lncRNAs was extracted from the TCGA database. The m6A-related lncRNAs were defined as lncRNAs that were significantly related to greater than or equal to one of the 23 m6A genes (|Pearson R| > 0.4 and p < 0.001). Finally, the m6A-lncRNA coexpression network was visualized using the Sankey diagram (**Figure 2A**), and 464 lncRNAs were discerned as m6A-related lncRNAs. The correlation between m6A genes and m6A-related lncRNAs of the model in the entire TCGA set is shown in **Figure 2B**.

### Construction and Validation of a Risk Model According to m6A-Related lncRNAs in KIRC Patients

**Figure 3:**
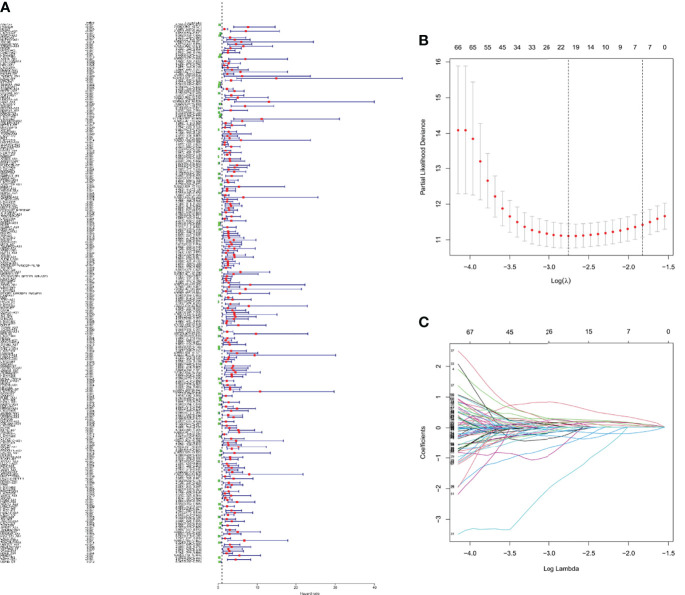
The risk model for KIRC patients based on m6A-related lncRNAs **(A)** Univariate Cox regression analysis revealed that the selected lncRNAs significantly correlated with clinical prognosis. **(B)** The tuning parameters (log λ) of OS-related proteins were selected to cross-validate the error curve. According to the minimal criterion and 1-se criterion, perpendicular imaginary lines were drawn at the optimal value. **(C)** The LASSO coefficient profile of 19 OS-related lncRNAs and perpendicular imaginary line were drawn at the value chosen by 10-fold cross-validation.

Univariate Cox regression analysis was adopted to screen m6A-related prognostic lncRNAs from 2876 m6A-related lncRNAs in the KIRC dataset from the TCGA database. In the TCGA database, 253 m6A-related lncRNAs were significantly associated with OS (**Figure 3A**). LASSO-penalized Cox analysis is commonly used for multiple regression analysis. It can not only enhance the prediction accuracy and ability of the statistical model, but also make variable options and regularization simultaneously. This method is extensively applied to the optimal choice of characteristics in high-dimensional data with an inferior correlation and prominent predicted value to avoid overfitting. Consequently, this method can effectively discern the most available prediction markers and produce a prognostic indicator to predict the clinical results. The dashed perpendicular line illustrates the first-rank value of log l with the minimum segment likelihood bias.Therefore, 19 m6A-related lncRNAs were selected for subsequent multivariate analysis (**Figures 3B, C**). Next, multivariate Cox ratio hazard regression analysis was performed to distinguish autocephalous prognostic proteins. 10 m6A-related LncRNAs were prognostic proteins independently correlated with OS in the training set and were used to construct a risk model to assess the prognostic risk of patients with KIRC (**Table S2**).

**Figure 4:**
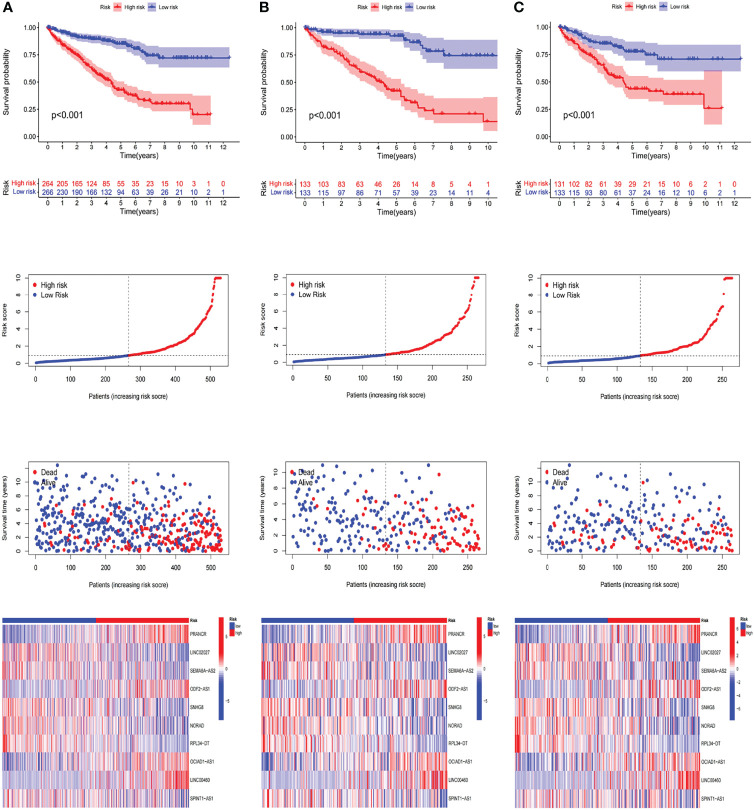
Prognostic value of the risk patterns of the m6A-related lncRNAs model in the TCGA dataset. (A1)Kaplan-Meier survival curves of the OS of patients in the high- and low-risk groups of the entire dataset (A2) Distribution of the m6A-related lncRNAs model-based risk score. (A3) Different patterns of survival status and survival time between both groups. (A4) The expression standards of the m6A-related lncRNAs model for each patient presented by the clustering analysis heatmap. **(B, C)** Relevant results of the train set and the test set.

According to the median of prognostic risk levels, KIRC samples were divided into the low-risk and high-risk groups, which were subjected to Kaplan-Meier survival analysis. **Figures 4A–C** present the survival status of patients in both groups in the whole dataset, the train set and the test set, respectively. The results indicated that the high-risk group had a poorer prognosis than the low-risk group, with a significant difference (P<0.001). **Figure 4A2** presents the distribution of risk levels of patients in both groups, and **Figure 4A3** presents the survival status and survival time of patients in both groups. The relative expression standards of the 10 m6A-related lncRNAs for each patient are shown in **Figure 4A4**.

**Figure 5:**
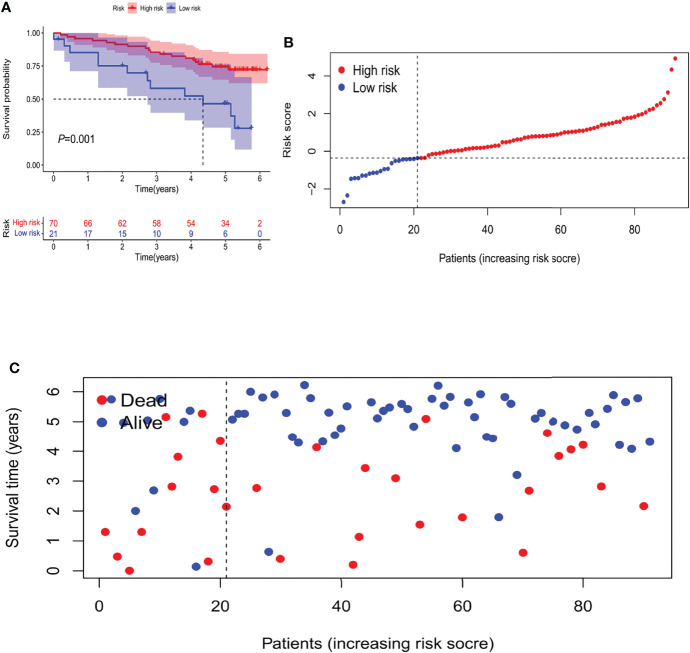
The Validation of Independent Cohort. **(A)** Kaplan-Meier survival curves of the OS of patients in the high- and low-risk groups. **(B)** Distribution of m6A-related lncRNA model-based risk score for the Validation set. **(C)** Patterns of the survival time and survival status between both groups for the Validation set.

In an attempt to test the prognostic capability of this established model, the risk scores of every patient in the train set and the test set were calculated with a uniform formula. **Figure 4B, C** presents the distribution of risk grades, the pattern of survival status and survival time, as well as the expression of the m6A-related lncRNAs in the train set (**Figures 4B1–B4**) and test set (**Figures 4C1–C4**). To further verify the accuracy and practicability of the model, we validated the expression profile data of 91 renal cell carcinomas in the ICGC database. The results show that the model still has a good effect on predicting survival time (**Figure 5**).

**Figure 6:**
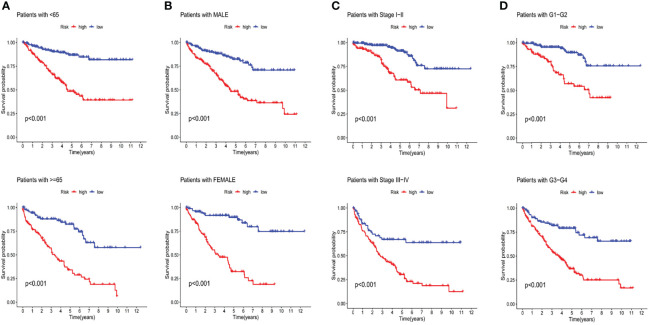
Kaplan-Meier curves of OS differences stratified by age **(A)**, gender **(B)**, stage **(C)** and tumor grade **(D)** between both groups in the entire TCGA set.

The discrepancies in OS stratified by the universal clinicopathologic features were analyzed between the low-and high-risk groups in the entire TCGA set. According to the subgroups classified by age, gender, stage and grade, the OS of the low-risk group continued to be superior to that of the high-risk group (**Figure 6**).

### Validation of the Grouping Ability of the m6A-Related lncRNA Model by PCA

**Figure 7:**
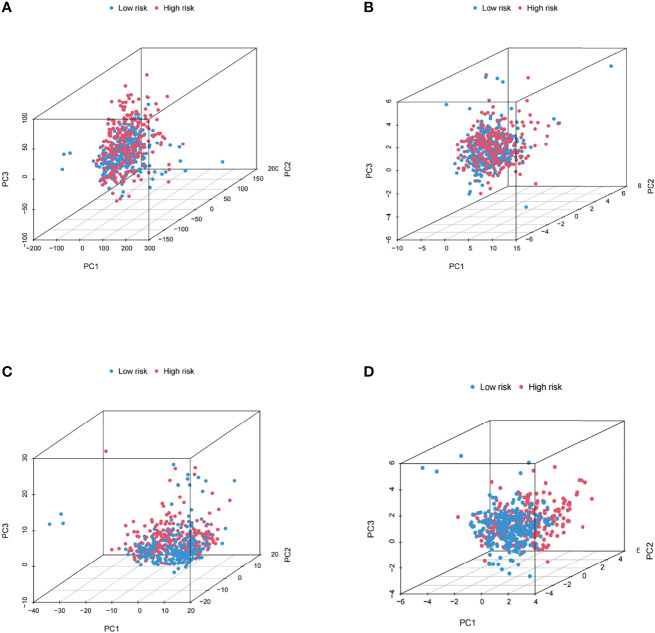
Principal component analysis between both groups based on **(A)** the entire gene expression profiles, **(B)** 23 m6A genes, **(C)** the m6A-related lncRNAs, and **(D)** the risk model based on the representation profiles of the 10 m6A-related lncRNAs in the entire TCGA set.

The PCA of this model was conducted to validate the difference between the low-risk and high-risk groups based on the entire gene expression profiles, 23 m6A genes, 464 m6A-related lncRNAs, and the risk model classified by the expression profiles of the 10 m6A-related lncRNAs (**Figures 7A–D**). As shown in **Figures 7A–C**, the distribution of the high- and low-risk groups is relatively scattered. However, the results obtained based on this model demonstrated that there were differences in the distribution between both groups (**Figure 7D**). These results suggested that the prognostic signature can be distinguished between both groups.

### Validation of the Grouping Ability of the m6A-Related lncRNA Model by PCA

The PCA of this model was conducted to validate the difference between the low-risk and high-risk groups based on the entire gene expression profiles, 23 m6A genes, 464 m6A-related LncRNAs, and the risk model classified by the expression profiles of the 10 m6A-related LncRNAs (**Figures 7A–D**). As shown in **Figures 7A–C**, the distribution of the high- and low-risk groups is relatively scattered. However, the results obtained based on this model demonstrated that there were differences in the distribution between both groups (**Figure 7D**). These results suggested that the prognostic signature can be distinguished between both groups.

### Evaluation of Tumor Immune Microenvironment and Tumor Immunotherapy Response by the m6A-Related lncRNA Model

**Figure 8:**
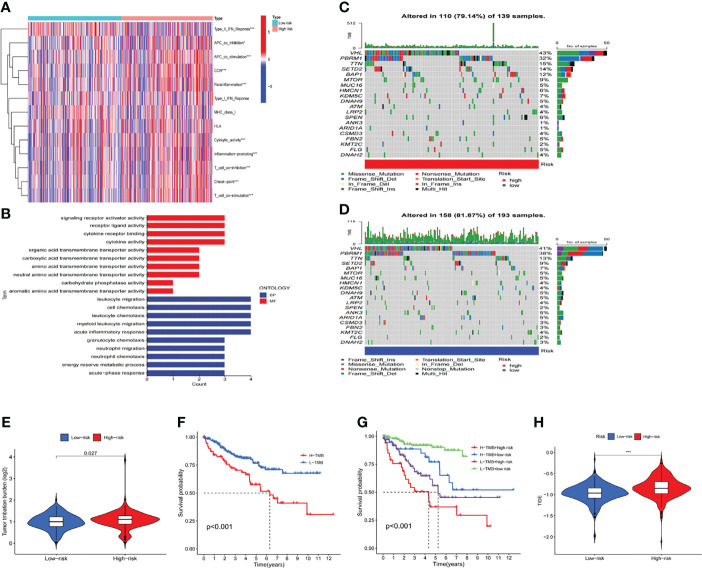
Estimation of the tumor immune microenvironment and cancer immunotherapy response with the m6A-related LncRNA model in the entire TCGA set **(A)** The indicated standards of the immunity index for each patient. **(B)** GO enrichment analysis. **(C, D)** The mutation information of the genes with high mutation frequencies in **(C)** the high-risk group and **(D)** the low-risk group presented by Waterfall plot. **(E)** TMB difference in patients of both groups. **(F, G)** Kaplan-Meier curve analysis of OS of patients classified according to the high/low mutation status and m6A-related lncRNA model. **(H)** TIDE prediction difference in patients of both groups.

The m6A-related lncRNA model was adopted to further analyze the enrichment level and activity of various immune cells, immune pathways or immune functions in 530 KIRC patients. There were significant differences in the expression of most immune indexes between both groups (**Figure 8A**). In an attempt to explore the potential molecular mechanism of the m6A-related lncRNA model, Gene Ontology (GO) enrichment analysis was conducted. The results of the BP group showed that the model molecules correlated with many immune-related biological processes (**Figure 8B**). Subsequently, the correlation between the m6A-related lncRNA model and immunotherapy biomarkers was explored. As expected, it was found that the high-risk group was more likely to respond to immunotherapies than the low-risk group, which indicated that this m6A-related lncRNA model can be used as an indicator to predict tumor immune dysfunction and exclusion (TIDE) (**Figure 8H**). In addition, the mutation data were analyzed and summarized by the R package maftools. The mutation was stratified according to the predictors of mutation effects. **Figures 8C, D** present the top 20 driver genes with the highest changing frequency between both subgroups. Then, the tumor mutation burden (TMB) scores were calculated based on TGCA somatic mutation data. The results suggested that the TMB score of the high-risk group was higher than that of the low-risk group, which indicated a high correlation between the m6A-related lncRNA model and TMB (**Figure 8E**). Moreover, Kaplan-Meier survival analysis of TMB was performed in tumor samples. The results in **Figure 8F** suggested that the high-mutation group had a poorer survival prognosis than the low-mutation group. As per further analysis, it was found that the high-mutation and high-risk group had the worst prognosis, while the low-mutation and low-risk group had a better prognosis. When the two groups had the high-mutation or low-mutation risk, the high-risk group still had a worse prognosis than the low-risk group (**Figure 8G**). These findings were also consistent with our previous results, which suggested that this risk model was effective and stable.

### Evaluation of the Prognostic Risk Model of m6A-Related LncRNAs and Clinical Features of KIRC

**Figure 9:**
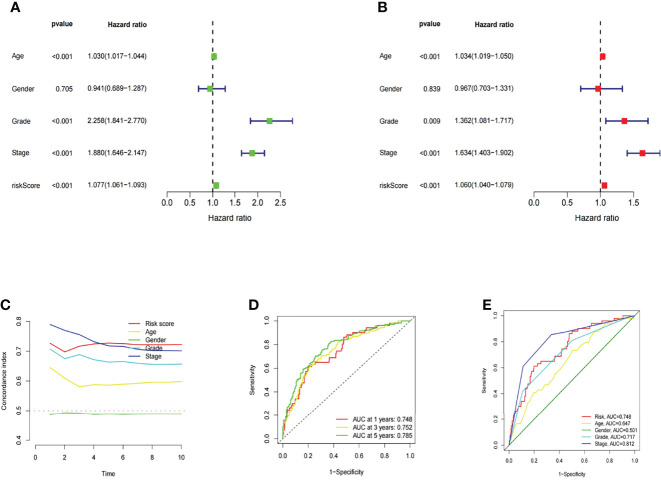
Assessment of the prognostic risk model of the m6A-related lncRNAs and clinical features of KIRC in the entire TCGA set. **(A, B)** Univariate and Multivariate analyses of the clinical features and risk scores with the OS. **(C)** Concordance indexes of the risk scores and clinical features. **(D, E)** ROC curves of the clinical features and risk scores.

In this study, univariate and multivariate Cox regression analyses were conducted to evaluate whether the risk model related to 10 m6A-related lncRNAs had independent prognostic characteristics of KIRC. The univariate COX regression analysis results showed that the odds ratios of HR and 95% CI were 1.077 and 1.061-1.093 (P<0.001), respectively (**Figure 9A**). The multivariate Cox regression analysis results showed that HR was 1.060 and 95% CI was 1.040-1.079 (P<0.001) (**Figure 9B**). It suggested that the risk model related to 10 m6A-related lncRNAs can effectively predict the prognosis independent of other clinical features. The concordance index and the area under ROC curve (AUC) of risk score were assessed to properly evaluate the uniqueness and sensitivity of risk score in predicting the prognosis of KIRC patients (**Figures 9D, E**). With the extension of time, the concordance index of risk score gradually increased with the risk level and became higher than that of other clinical factors. It suggested that the risk level of this model was effective in predicting the prognosis of KIRC patients (**Figure 9C**). The AUC of the risk level also became higher than that of most other clinicopathological factors. It suggested that 10 m6A-related lncRNAs can be reliably applied in the prognostic risk model for KIRC patients.

### Construction and Evaluation of the Prognostic Nomogram

**Figure 10:**
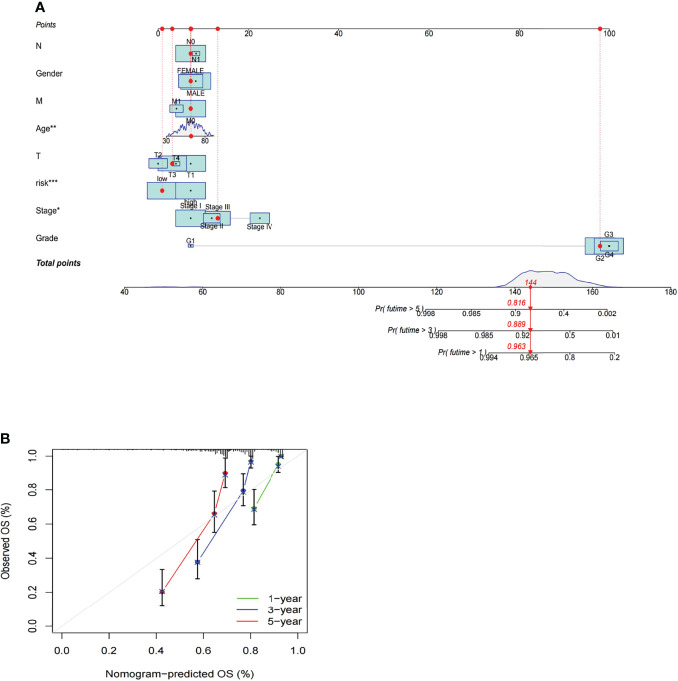
Construction and evaluation of a prognostic nomogram. **(A)** The likelihood of the 1-, 2-, and 3-year OS predicted by the nomogram. **(B)** The likelihood of the 1-, 2-, and 3-year OS predicted the calibration plot of the nomogram.

A nomogram incorporating the risk levels and clinical risk features was constructed to predict the OS of patients at 1, 2 and 3 years. According to the nomogram, the risk level of the prediction model showed a significant predictive ability through a comparison with clinical factors (**Figure 10A**). Relevant diagrams showed that there was favorable concordance in the observation and prediction rates of OS at 1, 2 and 3 years (**Figure 10B**).

### Identification of New Candidate Compounds for the m6A-Related lncRNA Model

In order to identify potential drugs for this m6A-related lncRNA model in the treatment of KIRC patients, the pRophetic algorithm was adopted to estimate the treatment response based on the half maximal inhibitory concentration (IC50) of each sample provided in Genomics of Drug Sensitivity in Cancer (GDSC). A total of 115 compounds were identified and there were significant differences in estimated IC50 between both groups. Among these compounds, 40 compounds were more sensitive in the low-risk group and 75 compounds were more sensitive in the high-risk group. **Supplementary Figure 1** presents partial sensitive compounds.

## Discussion

Renal cell carcinoma (RCC) is a malignancy from renal tubular epithelium, and its incidence ranks third among all tumors in the urinary system, with an upward trend with each passing year (26). Although surgical resection is the most effective method to treat RCC, the majority of patients have progressed to the middle and advanced stages at the moment of diagnosis. Besides, such tumors are not sensitive to radiotherapies, chemotherapies and immunotherapies, and short-term drug resistance may occur during the application of targeted therapies. Thus, RCC patients usually have a poor prognosis (27, 28). The occurrence and development of RCC are affected by multiple factors, and it is also a tumor related to multiple genes. Abnormal changes of gene expression regulatory network are also one of the important causes of RCC (29). The regulation of gene expression is affected by the gene level, transcription level and translation level, and the degradation of transcription products and protein products also determines the gene expression level. The identification of ncRNAs and their function provide novel insights for understanding the regulation of gene expression.

As per the systematic analysis of lncRNA expression profile, there are many abnormally expressed lncRNAs in RCC (30, 31), which could cause changes in protein expression and function and corresponding cell signaling pathways. Additionally, these abnormally expressed lncRNAs closely correlate with the occurrence, development, diagnosis, prognosis and drug resistance of RCC, such as metastasis-associated lung adenocarcinoma transcript 1 (MALAT1) with carcinogenic effects (32, 33), differentiation and antagonist ncRNA with cancer-suppressing effects (34), NONHSAT123350 related to the long-term survival rate of patients (27). These lncRNAs mainly interact with various RNA molecules and proteins in cis-action or trans-action mode in RCC, participate in histone modification, and regulate gene expression at the transcriptional level, post-transcriptional level and epigenetic level. Compared with other smaller non-coding RNAs, lncRNAs have a longer sequence and complex spatial structure, and they can play diverse and complex roles in gene regulation mechanisms. As an important modification process for RNA molecules including ncRNAs, m6A mainly affects lncRNAs through two regulatory mechanisms. For one thing, m6A can induce the binding of RNA binding proteins by providing binding sites for reader proteins or regulating local RNA structures. For another, m6A may also regulate the relationship between lncRNAs and specific DNA sites by affecting the RNA-DNA triple helix structure (35). As is reported in a recent study (36), METTL14 could affect the progression of ccRCC *via* the “METTL14-YTHDC1-Lnc-LSG1” regulatory axis. Besides, according to a study (37) of Gu et al., lncRNA DMDRMR can bind to IGF2BP3 and enhance the activity of IGF2BP3 by the m6A-dependent manner in KIRC, which would stabilize the expression of target genes CDK4, COL6A1, LAMA5 and FN1 and promote the G1/S transition of RCC.

However, there are insufficient studies on the pathological role of m6A and the role of lncRNAs in the progression of KIRC. In addition, there are scarce efforts to explore the biological mechanism and prognostic biomarkers of m6A-related lncRNAs related to KIRC. In this study, an independent prognostic model based on m6A-related lncRNAs is constructed based on the role of m6A and lncRNAs in KIRC. Further, the potential effective drugs for treating KIRC are also investigated based on this model. A total of 464 m6A-related lncRNAs are identified from the TCGA database, with a view to exploring the prognostic function of m6A-related lncRNAs. As per the results from the TCGA database, the prognostic value of 19 m6A-related lncRNAs is validated, including 10 that can be employed to construct the m6A-related lncRNA model to predict the OS of KIRC patients. Moreover, KIRC patients are divided into the high-risk group and the low-risk group according to the median of prognostic risk levels. The results indicate that the high-risk group has a poor prognosis. As per the multivariate Cox regression analysis results, the m6A-related lncRNA model is an autologous risk factor for OS. The ROC analysis results suggest that this model is more effective than most conventional clinical features in predicting the OS of KIRC patients. Furthermore, a nomogram is also plotted to present the perfect concordance between the observation and the prediction rates of the operating system at 1, 3 and 5 years. Finally, there is excellent concordance in the prediction rates of the operating system at 1, 3 and 5 years. The risk model based on 10 m6A-related lncRNAs independently related to OS has a higher accuracy, and this prediction model can be employed to identify new biomarkers for subsequent research.

TMB is a measure of the total amount of somatic coding mutations in a tumor, and it is related to the emergence of new antigens triggering anti-tumor immunity. As per recent studies, TMB is an effective biomarker for predicting the response to the therapy with PD-L1 (38). It can be found that the TMB of the low-risk group is lower than that of the high-risk group. Additionally, the TIDE algorithm is adopted to predict the likelihood of the immunotherapeutic response. The results indicate that the high-risk group has a larger immune response rate than the low-risk group, which also suggests that immune-related drugs may have better efficacy in the high-risk group in the prediction model. This finding also provides guidance values for the application of immune-related drugs.

As is known to all, pathological stage and grade are the decisive factors for the prognosis of KIRC patients. However, the same clinical stage and grade of tumors are not equal to the same prognosis. Therefore, it is of great significance to explore more comprehensive and specific predictive indicators or biomarkers. This m6A-related lncRNA model is constructed to provide a novel method for predicting the prognosis of KIRC patients. These findings also provide a new insight for exploring the modification process and mechanism of m6A in lncRNAs. In this study, multiple methods are adopted to verify this new model, and hence the optimal model can be properly selected and applied. Not only that, the validation of an external independent cohort in ICGC also suggests that the model has a good survival prediction ability.

However, there are still some defects and limitations in this study. The difference of cutoff values between the training set and validation set in the prognosis model may be a limitation that relatively limited the clinical practicability of the prognostic model (39). Moreover, it is also required to further verify the accuracy of this model through more external experiments, in an attempt to explore the role of lncRNAs and their interaction with m6A-related genes. In summary, the findings in this study provide novel insights for predicting the survival and prognosis of KIRC patients, which may contribute to revealing the process and mechanism of lncRNAs regulated by m6A. Furthermore, some potentially effective drugs are also preliminarily screened after constructing this immunotherapy-sensitive model, which brings some implications for the treatment of KIRC patients.

## Data Availability Statement

The datasets presented in this study can be found in online repositories. The names of the repository/repositories and accession number(s) can be found in the article/**Supplementary Material**.

## Author Contributions

Conception and design: DX and LB. Collection of clinical data and literature review: DX. Administrative, technical, or material support: QL, SY, and DX. Preparation of the manuscript: DX and LB. Revision of the manuscript: DX and LB. All authors contributed to the article and approved the submitted version.

## Funding

This work was supported by Clinical research cultivation program (2020lczd03) and the National Natural Science Foundation of China (81572107).

## Conflict of Interest

The authors declare that the research was conducted in the absence of any commercial or financial relationships that could be construed as a potential conflict of interest.

## Publisher’s Note

All claims expressed in this article are solely those of the authors and do not necessarily represent those of their affiliated organizations, or those of the publisher, the editors and the reviewers. Any product that may be evaluated in this article, or claim that may be made by its manufacturer, is not guaranteed or endorsed by the publisher.

## Supplementary Material

Supplementary Figure 1 Partial sensitive compounds.

Supplementary Table 1 The baseline characteristics of the training and testing set.

Supplementary Table 2 Model building.
