# Supplementary figures and images for: Construction of a Prognostic Model for KIRC and Identification of Drugs Sensitive to Therapies - A Comprehensive Biological Analysis Based on m6A-Related LncRNAs
Source: Front Oncol. 2022 Jun 2;12:895315. doi: 10.3389/fonc.2022.895315 (PMC9201082; doi:10.3389/fonc.2022.895315)

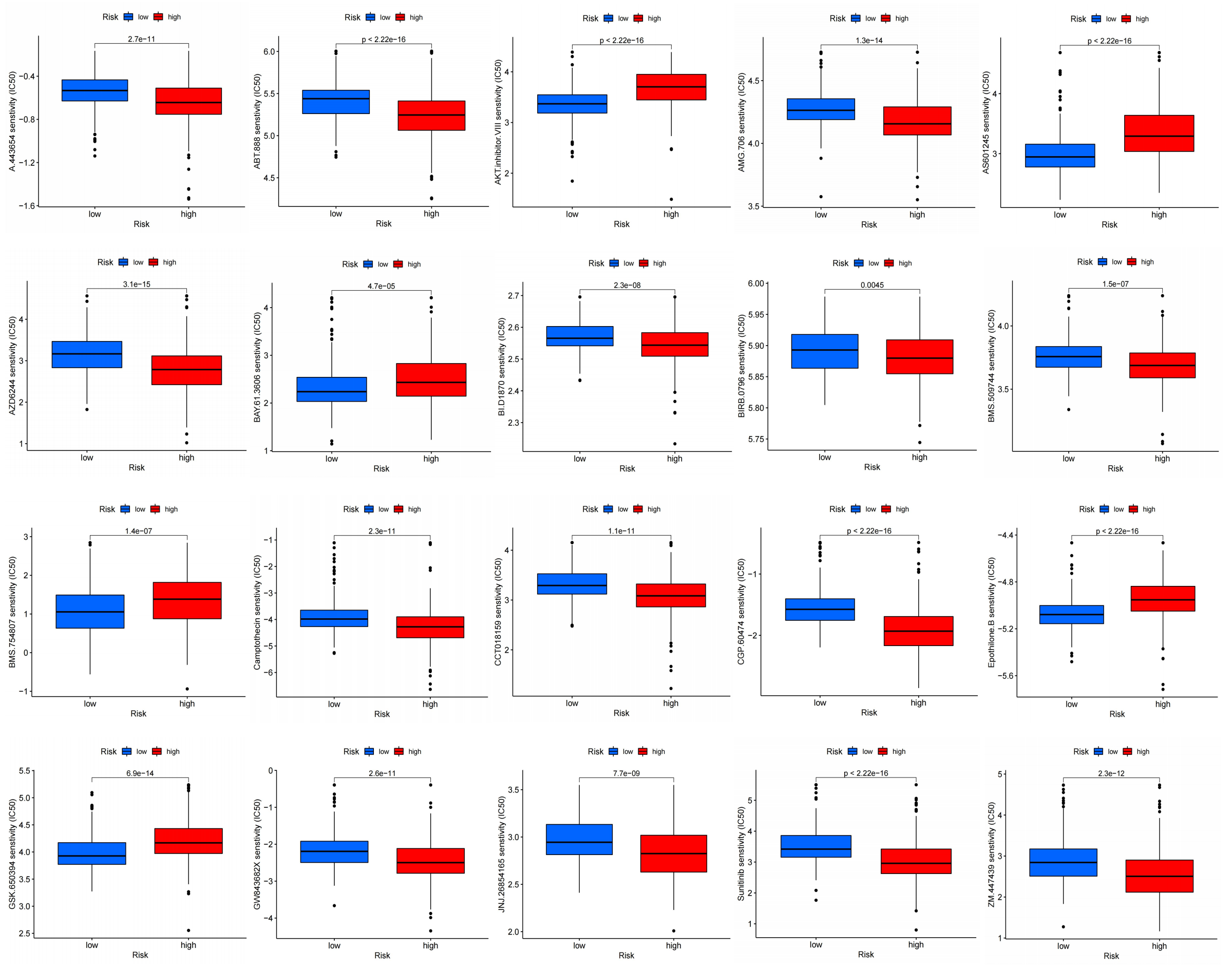

Supplement: Supplementary Figure 1 — Partial sensitive compounds. [file Image_1.tif]
